# Functional Study of the BMP Signaling Pathway in Appendage Regeneration of *Exopalaemon carinicauda*

**DOI:** 10.3390/biology14080940

**Published:** 2025-07-25

**Authors:** Chaofan Xing, Yong Li, Zhenxiang Chen, Qingyuan Hu, Jiayi Sun, Huanyu Chen, Qi Zou, Yingying Li, Fei Yu, Chao Wang, Panpan Wang, Xin Shen

**Affiliations:** 1 Jiangsu Key Laboratory of Marine Biotechnology, Jiangsu Ocean University, Lianyungang 222005, China; xingcf@jou.edu.cn (C.X.); lyde651@163.com (Y.L.); 18860810093@163.com (Z.C.); 18107246837@163.com (Q.H.); 13555073514@163.com (J.S.); mesace0719@163.com (H.C.); 13915285508@163.com (Q.Z.); yingyingli1247@163.com (Y.L.); 2 Jiangsu Key Laboratory of Marine Bioresources and Environment, Jiangsu Ocean University, Lianyungang 222005, China; 3 Co-Innovation Center of Jiangsu Marine Bio-Industry Technology, Jiangsu Ocean University, Lianyungang 222005, China; 4 Marine and Fishery Development Promotion Center of Lianyungang, Lianyungang 222000, China; yufei6@126.com (F.Y.); wangchao2698@163.com (C.W.); 5 The Jiangsu Provincial Infrastructure for Conservation and Utilization of Agricultural Germplasm, Nanjing 210014, China

**Keywords:** *Exopalaemon carinicauda*, limb blastema formation, BMP-mediated regeneration mechanism, transcriptome

## Abstract

Appendage autotomy frequently occurs during the cultivation of *Exopalaemon carinicauda*, which severely impacts its survival and economic benefits. To investigate the molecular mechanism underlying appendage regeneration in *E. carinicauda*, this study presents a comparative transcriptome analysis on samples from different stages of appendage regeneration in individuals of the same family of *E. carinicauda*. A total of 6460 differentially expressed genes (DEGs) were identified between the samples collected at 0 h post-autotomy (D0) and those collected at 18 h post-autotomy (D18h). Additionally, 7740 DEGs were identified between D0 and 14 d post-autotomy (D14d), with 3382 DEGs identified between D18h and D14d. Among them, differentially expressed genes such as *EcR*, *RXR*, *BMP1*, and *Smad4* are related to muscle growth or molting and may be involved in the regeneration process. qRT-PCR results revealed that *EcBMPR2* was expressed at relatively high levels in the gonad and ventral nerve cord tissues and that the highest level of expression was detected in the regenerative basal tissue at 24 h post-autotomy. In situ hybridization results indicated strong signals of this gene in the cells at the wound site at 72 h post-autotomy. Following knockdown of *EcBMPR2*, the expression levels of both *EcBMPR1B* and *EcSmad1* were significantly downregulated, and long-term interference with the *EcBMPR2* gene resulted in a significantly slower appendage regeneration process compared to the control group. When the downstream transcription factor *EcSmad1* was knocked down, the two receptor genes *EcBMPR2* and *EcBMPR1B* were downregulated, whereas *EcBMP7* was upregulated. After inhibiting the BMP signaling pathway, the degree of cell aggregation at the autotomy site in the experimental group was significantly lower than that in the control group, the wound healing rate was delayed, and the blastema regeneration time was prolonged from 5 d to 7 d. Collectively, these results indicate that the BMP signaling pathway plays a critical role in the early stages of appendage regeneration in *E. carinicauda*. This study provides important theoretical insights for understanding limb regeneration in crustaceans.

## Simple Summary

The regenerative mechanism underlying crustacean appendages stands as a critical area of research in biology. In this study, numerous genes involved in appendage regeneration in /Eriocheir carinicauda/ were identified. The results revealed that the *EcBMPR2* gene displayed the highest expression level in regenerative basal tissue at 24 h post-autotomy. In situ hybridization analyses indicated that this gene exhibited strong signals in cells adjacent to the wound site at 72 h post-autotomy. Long-term knockdown of genes within the BMP signaling pathway resulted in a slowdown of appendage regeneration, accompanied by a significantly reduced degree of cell aggregation at the autotomy site compared to the control group. These findings provide a theoretical foundation for elucidating the function of the BMP signaling pathway and the mechanism governing limb regeneration in crustaceans.

## 1. Introduction

Most aquatic crustaceans have the ability for appendage regeneration throughout their entire life cycle; the newly regenerated appendages can still regenerate after subsequent autotomy, and the structure and function of the regenerated appendages are well developed [1]. The molecular mechanism of regeneration is a complex biological process involving a series of physiological changes. In *Uca crabs*, appendage regeneration can only occur when the content of molting hormone is low [2,3]. It was found that appendage autotomy affects mating competition [4], and regeneration is influenced by nutritional factors and aquatic environmental physicochemical factors such as temperature and salinity [5]. The activin signaling pathway regulates the size of regenerated appendages in *Procambarus clarkii*, and inhibiting the key factor *Smox* can regenerate smaller yet intact appendages [6]. Regeneration in *Eriocheir sinensis* involves insulin-like and Hedgehog signaling pathways [7,8]. Complementary work by Wang et al. identified genes such as *Innexins* and *Slc7a5* that rapidly respond to autotomy and initiate regenerative responses [9]. While previous studies have identified regeneration-related genes such as *Wnt4* [10], *RXR* [11], and *EcR* [12] as being involved in different stages of the regeneration process in crustaceans, including *Eriocheir sinensis*, *Portunus trituberculatus*, and *Scylla paramamosain*, this study is the first to conduct a comprehensive functional analysis of the BMP signaling pathway in *Exopalaemon carinicauda*.

Bone morphogenetic proteins (BMPs), as signaling molecules that act locally on target cells, belong to the transforming growth factor β (TGF-β) superfamily. Originally identified as bone-inducing factors, they are now known to be involved in regulating the development of multiple organ systems, as well as cell survival, proliferation, and differentiation processes [13,14]. BMP signaling regulates proximodistal axis growth and dorsoventral patterning in vertebrate limb development [15]. While in *Branchiostoma*, blocking its pathway downregulates or ablates the expression of genes such as *Nodal* and *Lefty*, affecting the establishment of left-right asymmetry signaling [16]. The BMP signaling pathway influences abdominal morphology and gonopore development in *Portunus trituberculatus* by regulating the expression of *IAG* and *CFSH* [17]. Inhibition of the BMP signaling pathway in juvenile *Scylla paramamosain* reduces molting frequency and slows the growth rate [18]. Additionally, studies have found that BMPs are involved in biological regenerative processes. For example, in mammals such as mice, the BMP signaling pathway is essential for digit tip regeneration, and *BMP2/BMP7* can induce digit regeneration in non-regenerative proximal wounds [19,20]. Inhibition of the BMP signaling pathway slows the growth of regenerating limb buds and impedes tail regeneration in *Xenopus* tadpoles [21,22], whereas overexpression of axolotl *BMP2* induces limb abscission [23]. During tail regeneration in amphioxus, *BMP2/4* can regulate wound healing and blastema formation, while *Noggin* can inhibit this signaling, resulting in regeneration failure [24]. In the crustacean *Macrobrachium rosenbergii*, dysfunction of the BMP signaling pathway leads to prolonged wound healing time of appendages and impairs regenerative capacity [25]. During crayfish claw regeneration, BMP receptor genes contribute to early tissue regeneration by regulating stem cell proliferation and differentiation, while early high expression of *BMP2* promotes wound repair and initial blastema growth; conversely, late-stage upregulation of *BMP1* may be involved in structural reinforcement of the exoskeleton and musculature [6,26].

*Exopalaemon carinicauda* belongs to the genus *Exopalaemon* of the family *Palaemonidae*. With its short growth cycle and strong adaptability, it has become an economically important crustacean for coastal tidal flat and pond aquaculture in China [27,28,29,30]. With the increase in culture density, competition among *E. carinicauda* for limited resources such as food, living space, and breeding space intensifies, significantly affecting the growth performance, survival rate, and appendage integrity of *E. carinicauda* [31]. To investigate the molecular mechanisms underlying regeneration in *E. carinicauda*, this study integrated methods such as transcriptome sequencing, RNA interference, and histological analysis, and systematically elucidated the cascade regulatory network of BMP signaling pathway genes for the first time. These results not only fill the gap in understanding species-specific molecular mechanisms of regeneration but also provide a theoretical basis for research on limb regeneration in crustaceans.

## 2. Materials and Methods

### 2.1. Test Materials

Healthy *E. carinicauda* individuals were obtained from a full-sib family established by the Jiangsu Provincial Marine Biological Germplasm Resources Laboratory (Lianyungang, China). The average body length and weight of the shrimp were 4.9 ± 0.5 cm and 1.2 ± 0.4 g, respectively. The experimental water used was artificial seawater with a salinity of 25, pH of 8.1 ± 0.2, and a temperature of 18 ± 2 °C [32,33]. The shrimp were acclimated under well-aerated conditions for 7 d and fed commercial shrimp feed (Hailin, Xiamen, China) twice daily, with uneaten feed and feces removed promptly. One-third of the water was replaced every two days.

### 2.2. Collection of Experimental Samples

After the acclimation period, 300 healthy shrimp were randomly divided into two groups: a control group (normal group) and an experimental group (limb amputation group). Each group was set with 3 replicates, with 50 shrimp per replicate, and reared in a semi-static culture system within aquaculture tanks (80 cm × 50 cm × 30 cm). To reduce inter-individual interference, the tanks were partitioned into equal spaces using perforated plates (Fancychic, Jiangxi, China), with each shrimp occupying an independent compartment under identical rearing conditions.

For the experimental group, the left first and third walking legs and the right second and fourth walking legs were excised using the pressure method. In the control group, hepatopancreatic tissues were collected immediately after limb amputation. In the experimental group, hepatopancreatic tissues were collected at 18 h and 14 d post-amputation. Each sample was pooled from the hepatopancreatic tissues of 5 shrimp, immediately frozen in liquid nitrogen, and stored for total RNA extraction.

### 2.3. Total RNA Extraction and Synthesis of the First Strand of cDNA

Total RNA was extracted from hepatopancreatic tissue samples using RNAiso-Plus reagent (TaKaRa, Dalian, China). The integrity of RNA samples was assessed using an Agilent 2100 bioanalyzer (Agilent Technologies, Santa Clara, CA, USA), and a spectrophotometer was used to determine RNA purity and concentration, with detection indices including the RNA integrity number (RIN) and 28S/18S ratio. After quality assessment of the samples, 3 µg of total RNA was used for cDNA synthesis. Nine cDNA libraries were constructed using the NEBNext^®^ Ultra™ RNA Library Preparation Kit (NEB, Ipswich, MA, USA), including H0h_H1, H0h_H2, H0h_H3, H18h_H4, H18h_H5, H18h_H6, H14d_H7, H14d_H8, and H14d_H9.

### 2.4. Transcriptome Sequencing and Assembly

After passing library quality inspection, the qualified libraries were pooled according to their effective concentrations and target sequencing data volume requirements and then sequenced on an Ilumina NovaseqX Plus (Illumia, San Diego, CA, USA) platform. To ensure the quality and reliability of data analysis, the raw data were filtered by removing adapter-containing reads, N-containing reads, and low-quality reads to obtain high-quality clean data [34]. Clean reads from the nine sequencing libraries were assembled using Trinity software (version 2.6.6) to generate the unigene library of *E. carinicauda* [35]. The accuracy and integrity of the transcripts were evaluated using BUSCO software (v3.0.2). The assembled transcripts were annotated against the National Center for Biotechnology Information (NCBI)-nr, NCBI-nt, Protein family (Pfam), KEGG Orthology (KO), Gene Ontology (GO), and SwissProt databases to obtain functional information of the transcripts.

### 2.5. Analysis of Differentially Expressed Genes

Clean reads obtained from each sample were aligned to the assembled transcriptome using Bowtie2 (v2.2.5). Gene expression levels were quantified using RSEM (v1.2.12) and represented by FPKM (expected number of fragments per kilobase of transcript sequence per million base pairs sequenced) values. Differential expression analysis between groups was performed using the DESeq2 R package (1.20.0). The threshold for significantly differentially expressed genes was set as |log2 (fold change)| > 2 and adjusted *p*-value (*padj*) < 0.05 [31,36,37]. GO functional enrichment and KEGG pathway enrichment analyses of differentially expressed genes were conducted using GOseq (version 1.10.0) and KOBAS software (v2.0.12), respectively [37,38].

### 2.6. Real-Time PCR Experimental Validation

To verify transcriptomic data fidelity, 10 functionally annotated DEGs implicated in *E. carinicauda* development were selected for expression validation via qRT-PCR assays. cDNA samples were derived from the reverse transcription of RNA, which was the same batch of RNA used for preparing RNA-seq libraries. Meanwhile, a systematic analysis was conducted on the expression patterns of key genes in the BMP signaling pathway, such as *EcBMPR2*, in different tissues of *E. carinicauda* and in time-series samples of regenerated appendages after amputation. Quantitative primers were designed using Primer Premier 5.00 software (Premier Biosoft, Palo Alto, CA, USA) based on transcript sequences (Table S1) [39]. The amplification efficiency of the fluorescent quantitative PCR primers was determined using the SYBR^®^ Premix kit (TaKaRa, Dalian, China), and the specificity of the primers was assessed according to the melting curve.

### 2.7. Homologous Cloning and Sequence Analysis

Total RNA was extracted from the hepatopancreas of *E. carinicauda* using RNAiso-Plus reagent (TaKaRa, Dalian, China), and RNA quality was assessed. First-strand cDNA was synthesized using the PrimeScript™ II cDNA Synthesis Kit (TaKaRa, Dalian, China) according to the manufacturer’s instructions. Based on our transcriptome data, primer sequences (forward: 5′-TTTTTCTGCTGCTGTACCGG-3′, reverse: 5′-ATCACATCCCAACTGCCCTC-3′) were designed using Primer 5 software. The PCR amplification products were rapidly ligated into a TA vector (Sinomol, Nanjing, China), transformed into *E. coli* DH5α cells (Shenggong, Shanghai, China), and sequenced via paired-end sequencing. All open reading frames (ORFs) of the cloned gene were predicted using the ORF Finder (https://www.ncbi.nlm.nih.gov/orffinder/, accessed on 5 April 2025). Phylogenetic analysis was performed using MEGA software (v7.0, Allentown, PA, USA) [40].

### 2.8. In Situ Hybridization Analysis

*E. carinicauda* were subjected to limb amputation. Fresh tissue sections of the regenerative base were processed at 0 h, 72 h, and 14 d post-amputation. The sections were boiled in repair solution for 10–15 min, allowed to cool naturally, and then treated with proteinase K (20 μg/mL) at 37 °C for 10 min. After rinsing with pure water, the sections were washed three times with PBS for 5 min each. Permeabilization solution was applied to the tissue sections, which were allowed to permeate for 20 min. Afterward, the blocking agent was added, and the sections were incubated at room temperature in the dark for 15 min. The slides were then placed in PBS (pH 7.4) and washed three times on a shaking platform for 5 min each. The hybridization buffer was supplemented with 1% salmon sperm DNA mixture, mixed thoroughly, and applied to the sections. Prehybridization was conducted at 37 °C for 1 h. After the prehybridization mixture was discarded, the hybridization buffer containing a digoxigenin (Dig)-labeled oligonucleotide probe (probe sequence: 5′-AAGGGATTGGCGGTACAAGG-3′, with Dig modification at the 3′ end) was added, and the sections were hybridized overnight at 42 °C. After hybridization, the sections were washed, and mouse anti-DIG-labeled peroxidase (anti-DIG–biotin) was applied, followed by incubation at 37 °C for 1 h. HRP-conjugated streptavidin was then added, and the sections were incubated at room temperature for 15 min. TSA reagent (Ruisaiqi, Guangzhou, China) was applied for 30 min at 37 °C, followed by counterstaining with DAPI and mounting with coverslips. Images were captured using a Nikon fluorescence microscope (Nikon 80i, Tokyo, Japan), with all images acquired under consistent fluorescence capture settings for intensity and exposure time. Through the strength of the fluorescence signal, the qualitative analysis is performed to annotate gene-specific signals, primarily to explore their spatial distribution patterns.

### 2.9. Synthesis and Injection of Small Interfering RNA (siRNA)

Small interfering RNAs (siRNAs) targeting *EcBMP7*, *EcBMPR2*, *EcBMPR1B*, and *EcSmad1* were designed and synthesized using the T7 RNAi Kit (Vazyme, Nanjing, China) (Table S1). The synthesized siRNAs were quantified using a TGem Pro spectrophotometer. For the experimental groups, synthesized siRNAs were injected into the pericardial cavity of *E. carinicauda* at a dose of 6 μg/g body weight, whereas the control group received an injection of physiological saline. To further investigate the mechanism of action of the BMP signaling pathway, the highly potent BMP type I receptor inhibitor LDN-193189 tetrahydrochloride (MedChemExpress, South Brunswick Township, NJ, USA) was injected into the pericardial cavity at a dose of 15 μg/g body weight, with the control group receiving physiological saline injections. After injection, regenerated limbs were collected at the time point of maximal interference efficiency for qRT-PCR analysis. For each group, no fewer than 3 *E. carinicauda* were sampled, with three parallel groups set up, to investigate the interactions among BMP pathway genes in *E. carinicauda*.

The above injection procedure was repeated every 72 h, conducting a 12-day knockdown experiment with 80 *E. carinicauda* per group to observe limb regeneration, with no fewer than 5 individuals examined each time. Regenerative appendage samples were collected at 0 h, 24 h, 48 h, and 72 h post-treatment for section preparation and H&E staining. Histological and morphological observations were conducted to investigate the effects of the BMP signaling pathway on limb regeneration behavior.

### 2.10. Statistical Analysis

All qPCR data were derived from three biological replicates and three technical replicates. The 2^−△△Ct^ method was used to normalize the quantitative results with 18S rRNA used as the reference gene. One-way analysis of variance (ANOVA) was performed using IBM SPSS Statistics for Windows version 26.0 statistical software (IBM Corp., Armonk, NY, USA), and Duncan’s multiple comparison was used to test the significance of differences between means, with *p* < 0.05 indicating statistical significance.

## 3. Results

### 3.1. Transcriptome Assembly

Transcriptome sequencing and assembly generated a total of 19.3 Gb of data for the D0 library, including 66.58 million raw reads and 64.50 million clean reads. The D18h library produced 19.8 Gb of data, comprising 68.19 million raw reads and 65.78 million clean reads. For D14d, a total of 21.3 Gb of data were obtained, including 73.01 million raw reads and 70.90 million clean reads. A total of 55,376 unigenes were obtained, with an average length of 1135 bp and an N50 of 1839 bp. Species distribution analysis of the NR-annotated genes revealed that 63.9% of the genes were assigned to *Penaeus vannamei*.

### 3.2. Differential Gene Analysis

A total of 6460 DEGs were identified between the D0h and D18h samples, including 5008 upregulated genes and 1452 downregulated genes. Genes related to molting and growth, such as *insulin-like receptors*, *ecdysteroid-regulated-like proteins*, *ecdysis-triggering hormone receptors*, *SET and MYND domain-containing protein 4-like*, and *multiple epidermal growth factor (EGF)-like domains protein 8*, were enriched. A total of 7740 differentially expressed genes (DEGs) were identified between the D0h and D14d samples, including 5329 upregulated genes and 2411 downregulated genes. Among these DEGs, several putative muscle growth-related genes were detected, such as *actin 1*, *aurora kinase A-like isoform X2*, *tetraspan 33*, *leucine-rich repeat protein 1-like*, *ankyrin-1-like isoform X2*, and *myosin-IIIb-like*. A total of 3382 differentially expressed genes (DEGs) were identified between the D18h and D14d samples, including 1742 upregulated genes and 1640 downregulated genes. Several genes related to growth and immune stress, such as *suppressor of cytokine signaling*, *beta-glucuronidase-like*, *endothelial lipase-like*, *arylsulfatase B-like*, and *trypsin-1-like* genes, were identified. Moreover, the *mothers against decapentaplegic homolog 4-like* gene in the BMP signaling pathway presented significantly differential expression in the D0h vs. D18h comparison (Table 1).

### 3.3. Enrichment Analysis of Differentially Expressed Genes

To determine which biological processes and pathways are involved in the growth regulation of *E. carinicauda*, GO and KEGG analyses were performed on all differentially expressed genes (DEGs) across three comparison groups (D0h vs. D18h, D0h vs. D14d, D18h vs. D14d). The GO functional enrichment analysis showed that these DEGs were categorized into three types: molecular function, biological process, and cellular component. Figure S1 displays growth and development-related functional categories in each group, such as vesicle-mediated transport, mitotic nuclear division, transmembrane transport, and carbohydrate derivative metabolic process.

To better understand the functional characteristics of these DEGs, KEGG pathway enrichment analysis was performed on them. The DEGs were mapped to 295 KEGG pathways in the D0h vs. D18h group, 299 pathways in the D0h vs. D14d group, and 275 pathways in the D18h vs. D14d group. Figure S2 shows the significantly enriched pathways related to growth and development, such as Lysosome, cell cycle, and carbohydrate digestion and absorption.

### 3.4. Validation of RNA-Seq Results by qRT-PCR

**Figure 1:**
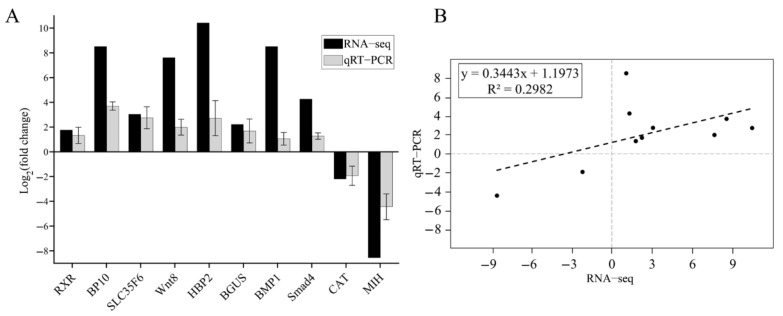
(**A**) Validation of fold changes of DEGs by RNA−Seq and qRT−PCR. RXR: retinoid X receptor; BP10: blastula protease 10−like isoform X1; SLC35F6: solute carrier family 35 member F6−like; Wnt8; HBP2: heme−binding protein 2; BGUS: beta−glucuronidase−like; CAT: cathepsin L; MIH: molt inhibiting hormone. (**B**) Regression analysis.

Ten growth and development-related DEGs were selected from the walking legs of *E. carinicauda* for qPCR analysis to validate the expression patterns of transcriptome data. These ten DEGs originated from different comparison groups: 18 h vs. 14 d (*RXR*, *MIH*, *BP10*), 0 h vs. 18 h (*CAT*, *SLC35F6*, *BMP1*, *Smad1*), and 0 h vs. 14 d (*WNT8*, *HBP2*, *BGUS*). qPCR results showed that all detected genes were upregulated between the two groups, except for CAT and MIH, which exhibited downregulation trends (Figure 1A). Regression analysis revealed that the fold changes in all genes assessed by both qPCR and RNA-seq were distributed in the first and third quadrants (Figure 1B). Overall, the expression trends of the same genes measured by qPCR and RNA-seq were consistent between the groups (Figure 1).

### 3.5. Structural and Phylogenetic Tree Analysis

The open reading frame (ORF) of the *BMPR2* gene was 2847 bp (GenBank accession number: PQ553568), encoding a total of 948 amino acids. Multiple sequence alignment results showed that the amino acid sequence of *EcBMPR2* shared the highest similarity (93%) with the sequences of *Macrobrachium rosenbergii* and *Macrobrachium nipponense* and exhibited 77% similarity with the sequence of *Halocaridina rubra* (Figure S3).

**Figure 2:**
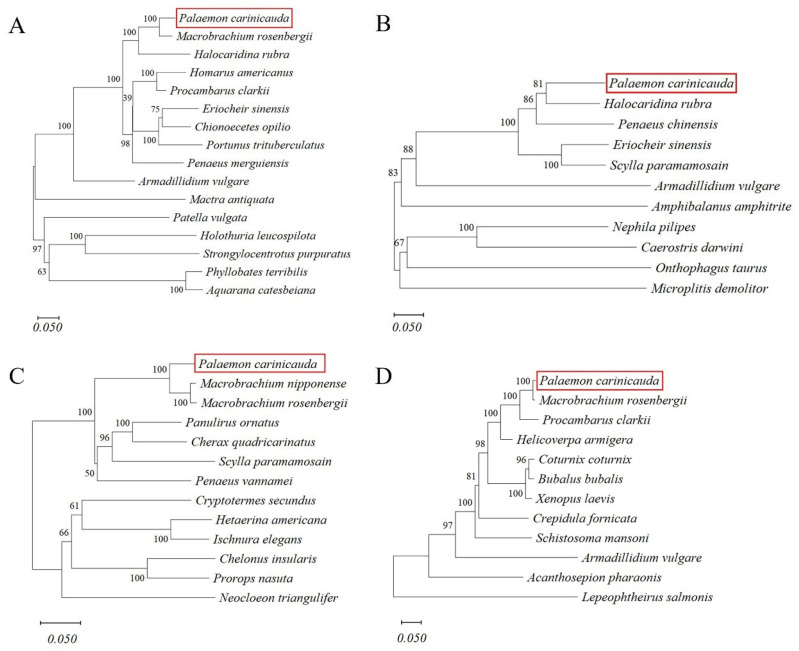
Phylogenetic analysis of key genes in the BMP signaling pathway. (**A**) Phylogenetic tree of *EcBMPR2*; (**B**) phylogenetic tree of *EcBMP7*; (**C**) phylogenetic tree of *EcBMPR1B*; (**D**) phylogenetic tree of *EcSmad1*. The *E. carinicauda* is marked with a red box in the figure.

Phylogenetic analysis revealed that the amino acid sequence of the *EcBMPR2* gene exhibited the closest relationship with *Macrobrachium rosenbergii*, first clustering with it, then forming a clade with *Halocaridina rubra*, and subsequently grouping with other chordates, including mollusks, echinoderms, and amphibians (Figure 2A). The amino acid sequence of the *EcBMP7* gene showed the closest relationship with *Halocaridina rubra* and first clustered with it, then formed a clade with *Penaeus chinensis* (Figure 2B). The amino acid sequence of the *EcBMPR1B* gene showed the closest relationship with *Macrobrachium nipponense* and first clustered with it, then formed a clade with *Macrobrachium rosenbergii* (Figure 2C). The amino acid sequence of the *EcSmad1* gene exhibited the closest affinity to *Macrobrachium rosenbergii*, subsequently clustering with *Procambarus clarkii*, and then grouping with other arthropods (Figure 2D).

### 3.6. Gene Expression During Regeneration

**Figure 3:**
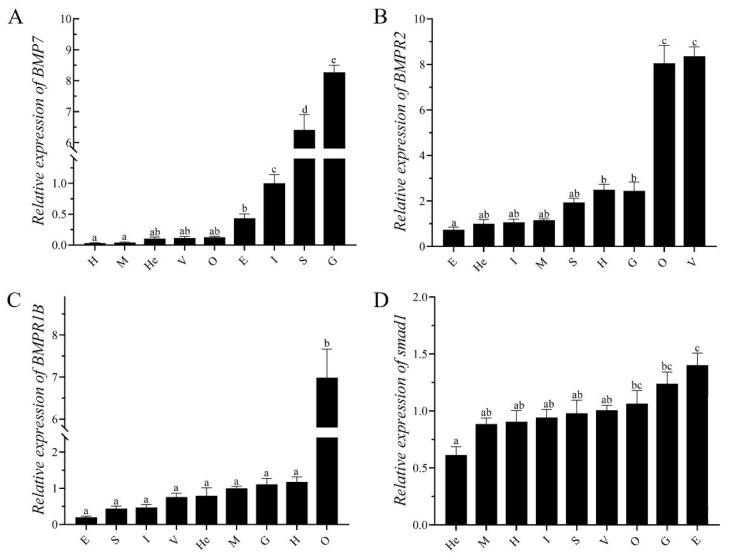
Tissue expression analysis of key genes in the BMP signaling pathway. (**A**) *EcBMP7*; (**B**) *EcBMPR2*; (**C**) *EcBMPR1B*; (**D**) *EcSmad1*. Different lowercase letters indicate significant differences (*p* < 0.05).

Tissue expression analysis of key genes in the BMP signaling pathway by qRT-PCR showed that the relative expression levels of *EcBMP7* mRNA varied across tissues. The gill tissue exhibited the highest relative expression level, followed by stomach and intestine tissues, whereas the heart and muscle tissues presented the lowest expression (Figure 3A). *EcBMPR2* showed higher expression levels in the ovary and ventral nerve cord than in other tissues, which are responsible for reproduction and nerve conduction, followed by the gills and heart, with the lowest relative expression in the eyestalk (Figure 3B). *EcBMPR1B* was significantly higher in ovary tissue than in all other tissues (Figure 3C), whereas *EcSmad1* exhibited the highest expression in the eyestalk and the lowest expression in the hepatopancreas (Figure 3D).

**Figure 4:**
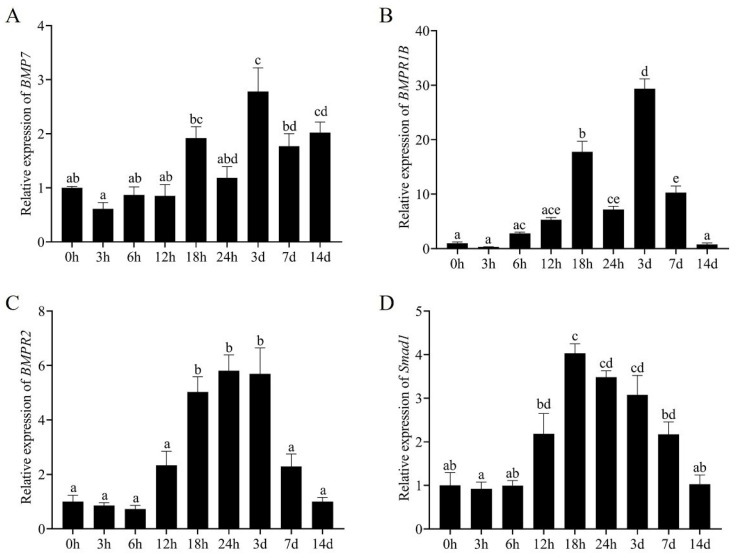
Expression analysis of key genes in the BMP signaling pathway during appendage regeneration. (**A**) *EcBMP7*; (**B**) *EcBMPR1B*; (**C**) *EcBMPR2*; (**D**) *EcSmad1*. Different lowercase letters indicate significant differences (*p* < 0.05).

Moreover, an analysis of the expression levels of BMP signaling pathway genes during appendage regeneration revealed that the expression of the *EcBMP7* gene gradually increased throughout the regeneration process. Its expression at 14 d remained significantly greater than that at 0 h, with no significant difference compared with that in the early regeneration stage (18 h) (Figure 4A). The *EcBMPR1B* gene was significantly expressed at the early regeneration stage (18 h), with its expression at 14 d being significantly lower than that at 18 h. The relative expression level peaked at 3 d, exhibiting an overall trend of first increasing but then decreasing (Figure 4B). The genes *EcBMPR2* and *EcSmad1* in the BMP signaling pathway tended to first increase but then slowly decrease. The expression levels of these genes began to rise rapidly 6 h after limb amputation, reached the highest relative expression at 18–24 h, and then gradually declined. By 14 d, their expression levels were not different from those at the time of amputation (0 h) (Figure 4C,D).

**Figure 5:**
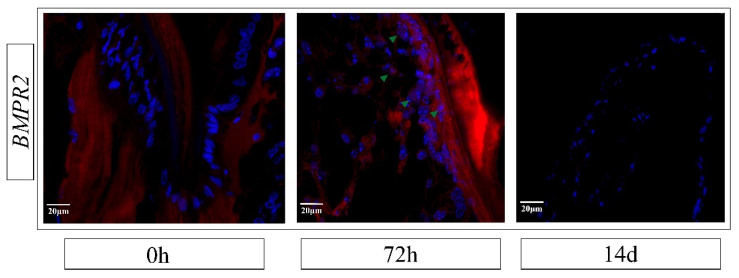
Expression localization of the *EcBMPR2* gene in basal tissue samples. Green arrows indicate specific expression signals, and blue staining represents cell nuclei. Bar: 20 μm.

Fluorescent in situ hybridization was performed using specific oligonucleotide probes for *EcBMPR2* to detect gene expression and localization in basal tissue samples of *E. carinicauda* appendages at 0 h, 72 h, and 14 d post-autotomy. At 0 h, no fluorescent signal for *EcBMPR2* was detected near the wound, which was covered by a layer of densely arranged cells. At 72 h post-amputation, *EcBMPR2* expression was detected in cells near the wound, localized in both the nucleus and cytoplasm. By late regeneration (14 d), the expression signal disappeared from the distal tip of the regenerating limb (Figure 5). These results align with the expression patterns of *EcBMPR2* observed in regenerating walking leg tissues at different stages, confirming temporal and spatial regulation of *EcBMPR2* during appendage regeneration.

### 3.7. Interactions Among BMP Signaling Molecules

**Figure 6:**
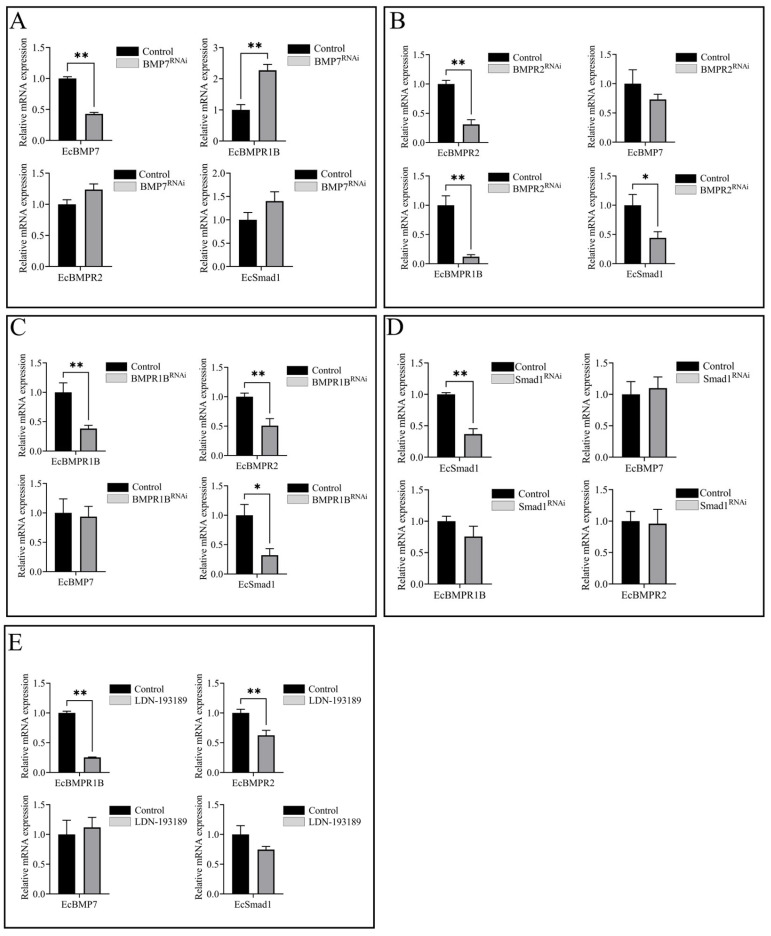
Regulatory interactions among BMP signaling molecules after appendage autotomy in *E. carinicauda*. (**A**) Injection of siRNA-*EcBMP7*; (**B**) injection of siRNA-*EcBMPR2*; (**C**) injection of siRNA-*EcBMPR1B*; (**D**) injection of siRNA-*EcSmad1*; (**E**) injection of LDN-193189. ‘*’ indicates significant difference (*p* < 0.05); ‘**’ indicates extremely significant difference (*p* < 0.01).

To investigate the regulatory mechanism of the BMP signaling pathway in limb regeneration of *E. carinicauda*, gene knockdown was performed via pericardial cavity injection of siRNAs targeting *EcBMP7*, *EcBMPR2*, *EcBMPR1B*, and *EcSmad1* after limb autotomy. After injection of siRNA targeting the *EcBMP7* gene, *EcBMP7* was significantly downregulated in basal muscle tissue of the appendage at 6 h, with the highest interference efficiency at this time. Conversely, the expression of the BMP type I receptor gene *EcBMPR1B* was significantly upregulated, and the expression of the BMP type II receptor *EcBMPR2* and downstream transcription factor *EcSmad1* were both upregulated, exhibiting an opposite trend as that associated with ligand knockdown (Figure 6A). As shown in Figure 6B, at 48 h after injection of siRNA targeting the *EcBMPR2* gene, *EcBMPR2* itself was significantly downregulated in basal muscle tissue of the appendage, with the highest interference efficiency observed at this time point. Additionally, the type I receptor *EcBMPR1B* and downstream transcription factor *EcSmad1* were both significantly downregulated. Although the expression level of the ligand *EcBMP7* showed a downward trend, it did not reach statistical significance. During the knockdown experiment of the *EcBMPR1B* gene, the highest interference efficiency was observed at 48 h post-injection, during which *EcBMPR1B* was significantly downregulated. The other receptor, *EcBMPR2*, and transcription factor *EcSmad1* were also significantly downregulated, whereas the expression of *EcBMP7* was not significantly different (Figure 6C). For knockdown of the downstream transcription factor *EcSmad1* in the pathway, maximum interference efficiency was achieved at 24 h post-injection, with *EcSmad1* significantly downregulated in the basal muscle tissue of the appendage. The two receptor genes were also downregulated, though not significantly; conversely, *EcBMP7* was not significantly upregulated at this time (Figure 6D).

To further investigate the interactions among signaling molecules in the BMP signaling pathway, the inhibitor LDN-193189 was injected for validation (Figure 6E). The results revealed that at 48 h post-injection, the type I receptor gene *EcBMPR1B* was significantly downregulated in the basal muscle tissue of the appendage, with the highest knockdown efficiency. The type II receptor gene *EcBMPR2* was also inhibited, and the expression level of the transcription factor *EcSmad1* decreased, whereas the ligand *EcBMP7* was upregulated. Based on the summarized trends of gene changes among groups (Table S3), we speculate that interactions exist among signaling molecules within the BMP signaling pathway in *E. carinicauda*. Furthermore, complex multi-level regulation likely occurs, and this requires further in-depth investigation.

### 3.8. Functional Studies of BMP Signaling Pathway Genes

**Figure 7:**
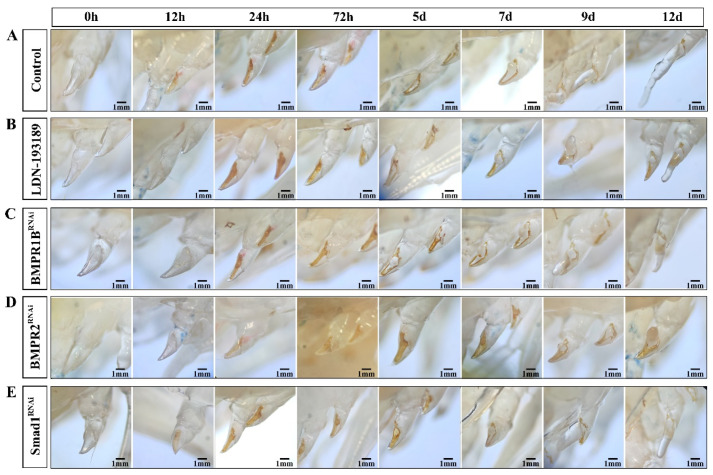
Effects of BMP signaling pathway knockdown on limb regeneration in *E. carinicauda*. (**A**) Saline injection (control); (**B**) injection of LDN-193189; (**C**) injection of siRNA-*EcBMPR1B*; (**D**) injection of siRNA-*EcBMPR2*; (**E**) injection of siRNA-*EcSmad1*. Bar: 1 mm.

Histological observations over 12 d showed that interference with key genes in the BMP signaling pathway affects the appendage regeneration process of *E. carinicauda* (Figure 7). The details are as follows: Immediately after limb amputation (0 h), the wound showed obvious swelling and was covered with a transparent film. At 12 h post-amputation, both the siRNA-*EcSmad1* group (Figure 7E) and the control group (Figure 7A) formed yellowish-brown scabs with no difference in healing, while the receptor-knockdown groups (Figure 7B–D) only showed pigmentation at the wound edges. Among them, the scab color in the siRNA-*EcBMPR2* group was significantly lighter than that in the control group at 24 h. During the critical period from 72 h to 5 d post-amputation, the scabs in the control group and other knockdown groups (except siRNA-*EcBMPR2*) faded and formed milky blastemas, while the siRNA-*EcBMPR2* group was severely delayed, with blastemas appearing only at 7 d post-amputation, and slowly developing into transparent limb buds after 9 d. By 12 d, the size of the regenerated limbs in all knockdown groups was smaller than that in the control group.

**Figure 8:**
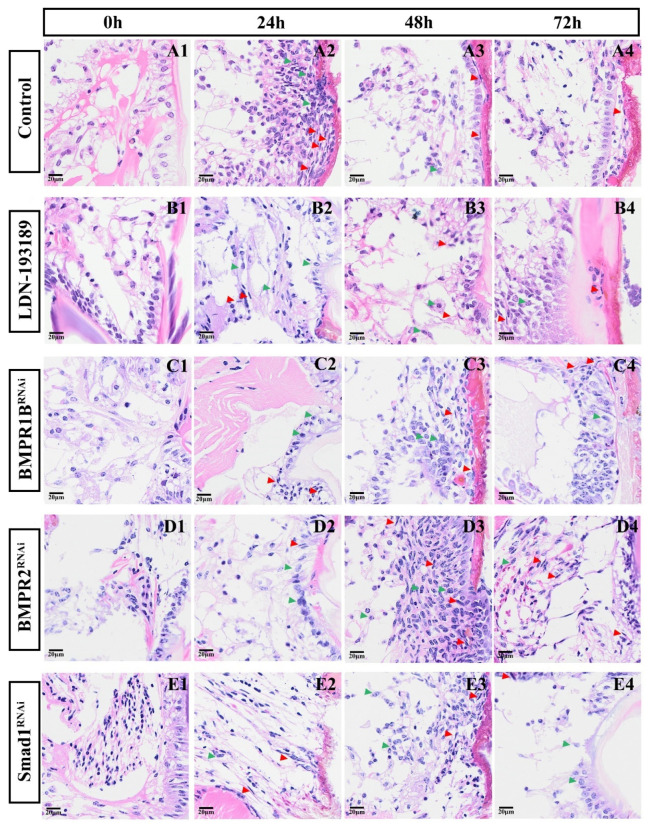
H&E staining of tissue sections after BMP signaling pathway knockdown. (**A1**–**A4**) Saline injection (control); (**B1**–**B4**) injection of LDN-193189; (**C1**–**C4**) injection of siRNA-*EcBMPR1B*; (**D1–D4**) injection of siRNA-*EcBMPR2*; (**E1–E4**) injection of siRNA-*EcSmad1*. Green arrows indicate granular cells, and red arrows indicate spindle-shaped cells. Bar: 20 μm.

To further understand the morphological changes in cell tissues in the early stage of inhibited appendage regeneration, H&E staining was performed based on the morphological observation experiment of long-term interference with key genes in the BMP signaling pathway affecting appendage regeneration, and the results are shown in Figure 8. At 24 h, the migration of cells to the wound in the knockdown groups was significantly reduced (most notably, in the siRNA-*EcBMPR2* group). At 48 h, the control group had formed a tight columnar epithelium, while none of the knockdown groups established an orderly structure (the siRNA-*EcBMPR2* group had the highest degree of cell aggregation). At 72 h, other knockdown groups began epithelial reorganization, while the siRNA-*EcBMPR2* group remained in a state of disordered aggregation of spindle-shaped cells.

## 4. Discussion

The regenerative mechanism of crustacean appendages represents a crucial area of research in biology. This process involves key biological phenomena such as cell proliferation, differentiation, apoptosis, and signal transduction. This study first conducted transcriptomic analysis on the hepatopancreas tissue of *E. carinicauda* after limb amputation, and found that immune-related genes such as *beta-glucuronidase-like*, *leucine-rich repeat protein 1-like*, and *endothelial lipase-like* were differentially expressed in the early stage of appendage regeneration, which are used to activate immune responses to prevent wound infection. Furthermore, growth-related factors involved in cell growth and differentiation, including *SMYD4*, *clavesin-2-like*, and *trypsin-1-like*, were identified, providing new molecular evidence for wound healing and limb development during the early stage of appendage regeneration [41,42,43,44]. In terms of the relationship between regeneration and molting, crustaceans possess an exoskeleton composed of chitin and cuticular proteins, which restricts the growth and development of muscles [45]. Previous studies have shown that limb amputation in *Scylla serrata* slows down growth and prolongs the molting cycle [46], while appendage regeneration in fiddler crabs occurs only when the ecdysone level is low [2]. In this study, we are the first to identify a close association between regeneration and molting-related genes such as *ECR*, *RXR*, *MIH*, *ETH* [47], and *Troponin C* [48] in *E. carinicauda*, further confirming a link between appendage regeneration and molting. Moreover, in the functional analysis of differentially expressed genes, several genes such as *HSP70*, *elongator complex protein 5-like*, and *decaprenyl-diphosphate synthase subunit 1-like* are involved in energy metabolism of carbohydrates, lipids, and amino acids. This clarifies the mechanism underlying the demand for additional energy during the regeneration process [49,50], supplementing molecular evidence for the regulation of regenerative energy metabolism in crustaceans.

The role of the BMP signaling pathway in regeneration is the core of this study. Although previous studies have involved BMP family genes in crustaceans such as *Eriocheir sinensis* and *Scylla paramamosain* [51,52], they have not thoroughly analyzed their specific functions in appendage regeneration. In this study, we used RNAi technology to knockdown key genes in the BMP signaling pathway. In the BMP receptor gene knockdown group, we observed that immediately after limb amputation (0 h), the wound was covered by a transparent membrane. By 12 h, pigment accumulation was only detected at the wound edges, and subsequent wound coloration progressed more slowly compared to the control group. These results suggest that BMP signaling pathway genes may participate in cell differentiation, thereby regulating wound healing after limb amputation in *E. carinicauda*. In the process of animal regeneration, the wound healing stage occupies a core position, creating favorable conditions for the subsequent stages of cell proliferation and morphogenesis [53]. Hopkins et al. divided the regenerative stages of crustacean appendages into five stages, including Scab Formation and Papilla Formation [46,54]. Studies on the regeneration of *Eriocheir sinensis* also showed that the papillary blastema is formed only after the wound is covered by melanized scabs [9,55]. Combined with H&E staining observations, it was found that at 24 h after limb amputation, the degree of cell aggregation in the control group was higher than that in the knockdown group. This directly corresponds to the result of delayed wound healing caused by BMP knockdown in morphological observations, providing morphological evidence for the regulation of early regenerative cell behaviors by BMP signaling. In addition, granular cells and spindle-shaped cells were found near the wound. Based on studies on *Macrobrachium rosenbergii* and our previous research on the process of appendage regeneration [25,31], it is speculated that these cells are crucial for wound healing, while their specific functions and mechanisms of action require further investigation.

When the wound scab begins to gradually fade and a papillary blastema forms, it indicates that the regenerating limb has entered a stage of rapid growth. BMP signaling can induce apoptotic signals; for example, the *BMP2* and *BMP4* genes can induce the apoptosis of undifferentiated cells in the early regenerating limb buds [56]. Beck et al. found that blocking BMP signaling resulted in the cessation of limb bud and tail regeneration in *Xenopus laevis* tadpoles [22]. In our study, after continuous knockdown of key genes in the BMP signaling pathway for 12 d, it was observed that the regeneration rate of limbs in *E. carinicauda* was slowed down and the regeneration efficiency was inhibited, which confirms that the BMP signaling pathway is indeed involved in the appendage regeneration process of *E. carinicauda*.

BMP belongs to the TGF-β superfamily and plays critical roles in embryonic development, cell proliferation, differentiation, and regeneration processes [57]. To further analyze the regulatory mechanism of the BMP pathway, this study investigated the expression patterns of key genes in the BMP signaling pathway of *E. carinicauda*. It was found that the detected BMP signaling pathway genes showed differential expression in various tissues: the ligand gene *EcBMP7* had a relatively high expression in gill tissues, while the receptors *EcBMPR2* and *EcBMPR1B* had relatively high levels in gonadal tissues. Cheifetz et al. reported that the *BMPR2* gene in *Danio rerio* is ubiquitously distributed and is expressed in various tissues [58]. Studies have shown that *BMP2*, *BMPR1B*, and *BMPR2* exhibit differential expression in the early stage of limb regeneration [25,26]. In the early development of mice, *BMPR2* is crucial for transducing BMP signals and serves as a key factor essential for ectodermal differentiation and mesodermal induction [59]. Similarly, in this study, during the process of appendage regeneration, the detected BMP signaling pathway genes were activated and their expressions were up-regulated in the early stage of regeneration. Meanwhile, through in situ hybridization, *EcBMPR2* signals were detected in the cells near the wound at 72 h after limb amputation. These findings indicate that the BMP signaling pathway may play a role in cell proliferation and differentiation, and is involved in the molecular regulation during the early stage of appendage regeneration in *E. carinicauda*.

Due to the mechanism of signal transduction, activation of the BMP signaling pathway requires BMP receptors. *BMPR2* serves as a specific type II receptor for the BMP family and remains constitutively active in the absence of a ligand [60]. In this study, the open reading frame (ORF) of *EcBMPR2* was cloned, spanning 2847 base pairs and encoding a total of 948 amino acids. When ligands bind to the receptor, type II receptors phosphorylate the GS domain of type I receptors, thereby regulating downstream signal transduction [61]. BMP ligands activate SMAD proteins and target genes by binding to two BMP receptors, while the TGF-β signaling pathway transduces signals from the extracellular environment to the nucleus through SMAD proteins [62,63]. In this study, it was found that after knocking down the *EcBMPR2* gene, both *EcBMPR1B* and *EcSmad1* were significantly down-regulated; meanwhile, after knocking down *EcBMPR1B*, *EcBMPR2* and *EcSmad1* were also significantly down-regulated. This phenomenon echoes the result in *Scylla paramamosain*, where silencing of *BMPR1B* leads to a decrease in *Smad1* expression [52], suggesting that a BMP-SMAD regulatory mechanism may also exist in *E. carinicauda*. Furthermore, experiments using the LDN-193189 inhibitor confirmed that it can suppress the expression of *EcBMPR1B*, *EcBMPR2*, and *EcSmad1*, which is consistent with the inhibitory effect observed in studies where LDN-193189 was used to inhibit the BMP signaling pathway in *Scylla paramamosain* [18]. What is more interesting is that in the knockdown experiment of *EcBMP7*, the absence of the ligand gene *EcBMP7* caused an upregulation of receptor genes in the short term. It is speculated that the BMP signaling pathway may maintain its physiological functions by increasing the number of receptors or interacting with other pathways. For example, *Activin A* can not only bind to the *AACVR2* but also to *BMPR2*, thereby promoting the ACVR signaling pathway [64]. However, such a speculation about this compensation mechanism has not been reported in previous studies on crustaceans, and further research is needed.

This study systematically reveals the molecular mechanism underlying the appendage regeneration process of *E. carinicauda*, and particularly clarifies that the BMP signaling pathway is closely related to both the initiation of regenerative behavior and the differentiation during the process. Among them, *EcBMPR2* plays a significant role in the wound healing stage during the early regeneration period. Studies have speculated that BMPs also participate in the appendage regeneration process of *E. carinicauda*; however, their mechanism of action and whether different regulatory patterns exist in various tissues still require in-depth investigation.

## 5. Conclusions

In the context of high-density aquaculture, the frequency of autotomy in crustaceans increases due to factors such as space constraints. For high-value crustaceans like crabs, limb loss can significantly affect their market value, while regenerative capacity enables them to recover quickly. This study demonstrates that BMP signaling, particularly via *EcBMPR2*, is crucial for early wound healing and appendage regeneration in *E. carinicauda*, providing a basis for further investigations into the molecular mechanisms of crustacean limb regeneration. Furthermore, our research is expected to provide information for the precision breeding of crustaceans, aiming to obtain aquaculture species with characteristics such as strong adaptability and rapid growth.

## Figures and Tables

**Table 1:** Genes related to growth and molting traits in *E. carinicauda*.

Groups	Gene ID	log2 (FC)	*padj*	NR Description
D0 and D18h	Cluster-10860.6202	5.9999	0.000	ecdysis triggering hormone receptor [*Macrobrachium nipponense*]
	Cluster-10860.5779	4.7732	0.000	insulin-like receptor [*Macrobrachium rosenbergii*]
	Cluster-10860.5018	4.4719	0.000	SET and MYND domain-containing protein 4-like [*Penaeus vannamei*]
	Cluster-10860.4916	2.9628	0.002	multiple epidermal growth factor-like domains protein 8 [*Penaeus vannamei*]
	Cluster-10860.26681	−4.2185	0.000	ecdysteroid-regulated-like protein [*Penaeus vannamei*]
	Cluster-10860.10375	4.270	0.000	mothers against decapentaplegic homolog 4-like [*Penaeus vannamei*]
D0h and D14d	Cluster-10860.33757	7.8190	0.000	myosin-IIIb-like [*Penaeus vannamei*]
	Cluster-10860.11667	6.6466	0.000	troponin C, isotype gamma-like isoform X1 [*Penaeus vannamei*]
	Cluster-10860.19895	5.9363	0.000	leucine-rich repeat protein 1-like [*Penaeus vannamei*]
	Cluster-10860.31481	3.6982	0.000	aurora kinase A-like isoform X2 [*Penaeus vannamei*]
	Cluster-10860.7806	3.5121	0.000	muscle M-line assembly protein unc-89-like [*Penaeus vannamei*]
	Cluster-10860.29146	2.7223	0.000	heat shock protein [*Penaeus monodon*]
	Cluster-10860.32350	1.8211	0.004	actin 1 [*Penaeus vannamei*]
	Cluster-10860.27491	−2.2381	0.001	tetraspan 33 [*Macrobrachium rosenbergii*]
	Cluster-10860.6902	−3.5287	0.000	ankyrin-1-like isoform X2 [*Penaeus vannamei*]
	Cluster-10860.8082	6.8777	0.000	blastula protease 10-like isoform X1 [*Penaeus vannamei*]
D18h and D14d	Cluster-10860.26453	7.4777	0.000	endothelial lipase-like [*Penaeus vannamei*]
	Cluster-10860.21651	4.8378	0.000	arylsulfatase B-like [*Penaeus vannamei*]
	Cluster-10860.22652	3.4228	0.000	beta-glucuronidase-like [*Penaeus vannamei*]
	Cluster-10860.18694	−2.4482	0.000	trypsin-1-like [*Penaeus vannamei*]
	Cluster-10860.15113	−3.9925	0.000	suppressor of cytokine signaling [*Penaeus vannamei*]

## Data Availability

The data included in this study can be provided on request from the corresponding authors.

## Supplementary Materials

The following supporting information can be downloaded at: https://www.mdpi.com/article/10.3390/biology14080940/s1.
