# Supplementary material for: Functional Study of the BMP Signaling Pathway in Appendage Regeneration of Exopalaemon carinicauda
Source: Biology (Basel). 2025 Jul 25;14(8):940. doi: 10.3390/biology14080940 (PMC12383973; doi:10.3390/biology14080940)
Supplement: Supplementary file 1 [file biology-14-00940-s001.zip › Figure S3.pdf]

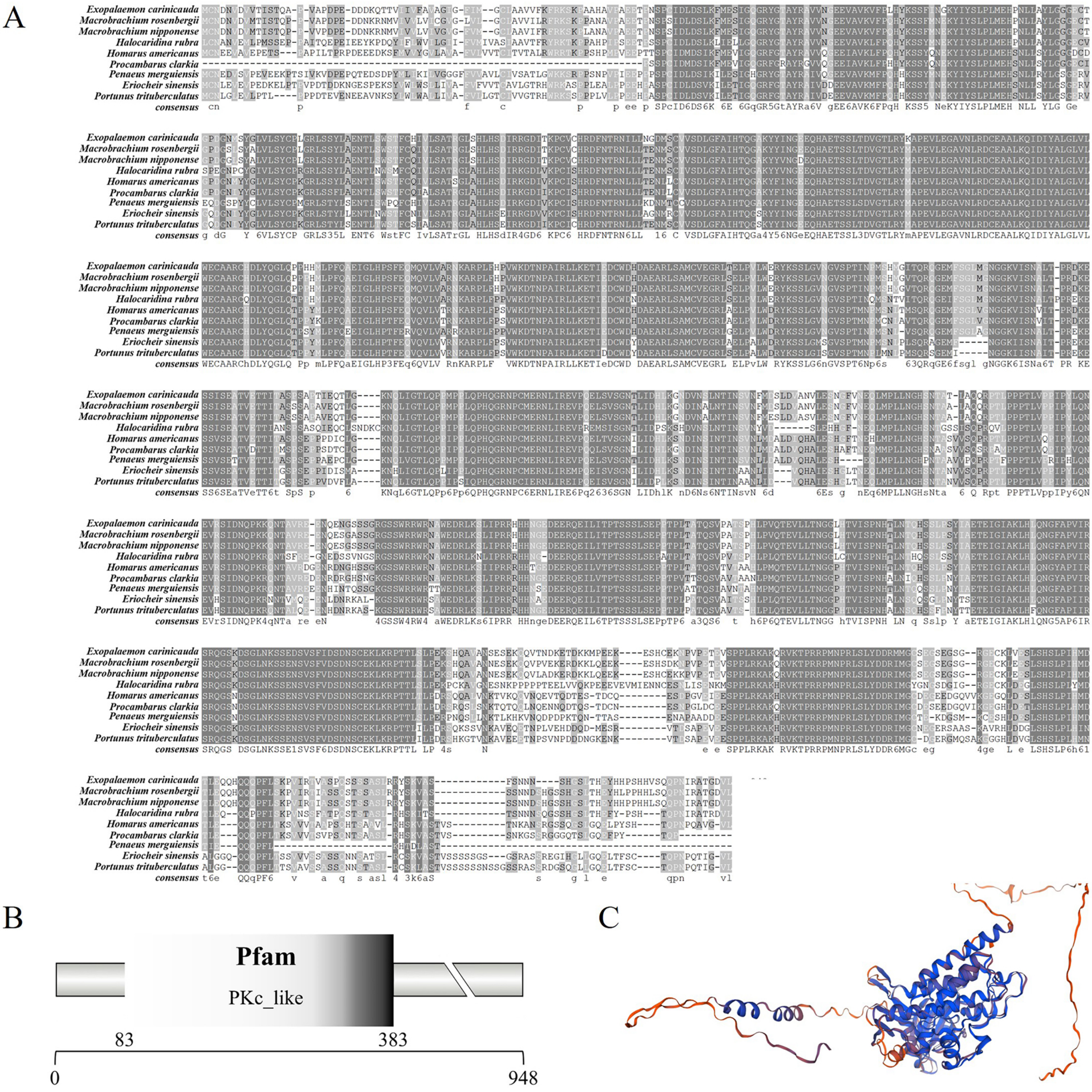
A
Exopalaemon carinicauda
Macrobrachium rosenbergii
Macrobrachium nipponense
Halocaridina rubra
Homarus americanus
Procambarus clarkia
Penaeus merguiensis
Eriocheir sinensis
Portunus trituberculatus
consensus
B
Pfam
PKc_like
83
383
0
948
C
