# Supplementary figures and images for: Functional Study of the BMP Signaling Pathway in Appendage Regeneration of Exopalaemon carinicauda
Source: Biology (Basel). 2025 Jul 25;14(8):940. doi: 10.3390/biology14080940 (PMC12383973; doi:10.3390/biology14080940)

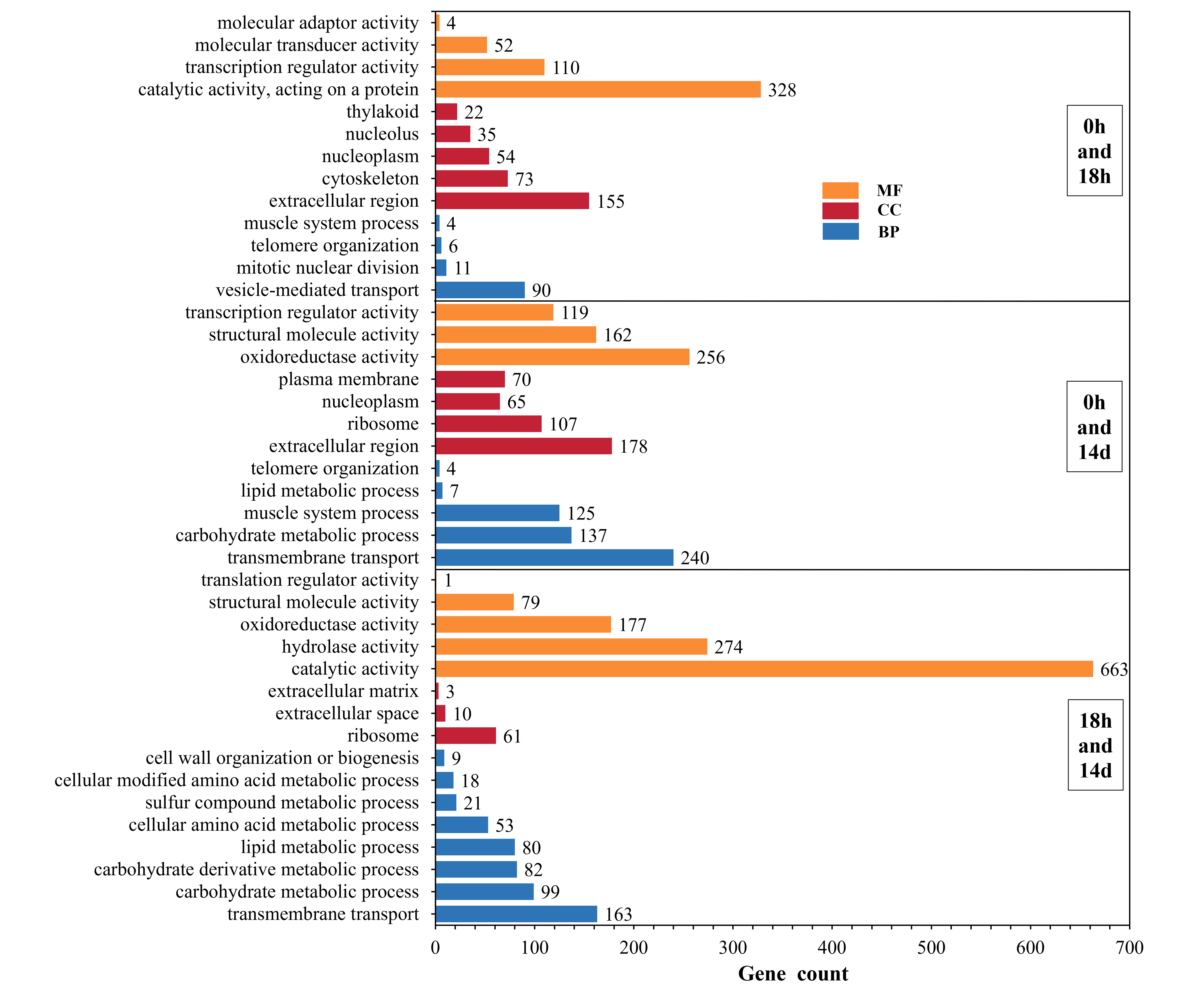

Supplement: Supplementary file 1 [file biology-14-00940-s001.zip › Figure S1.jpg]

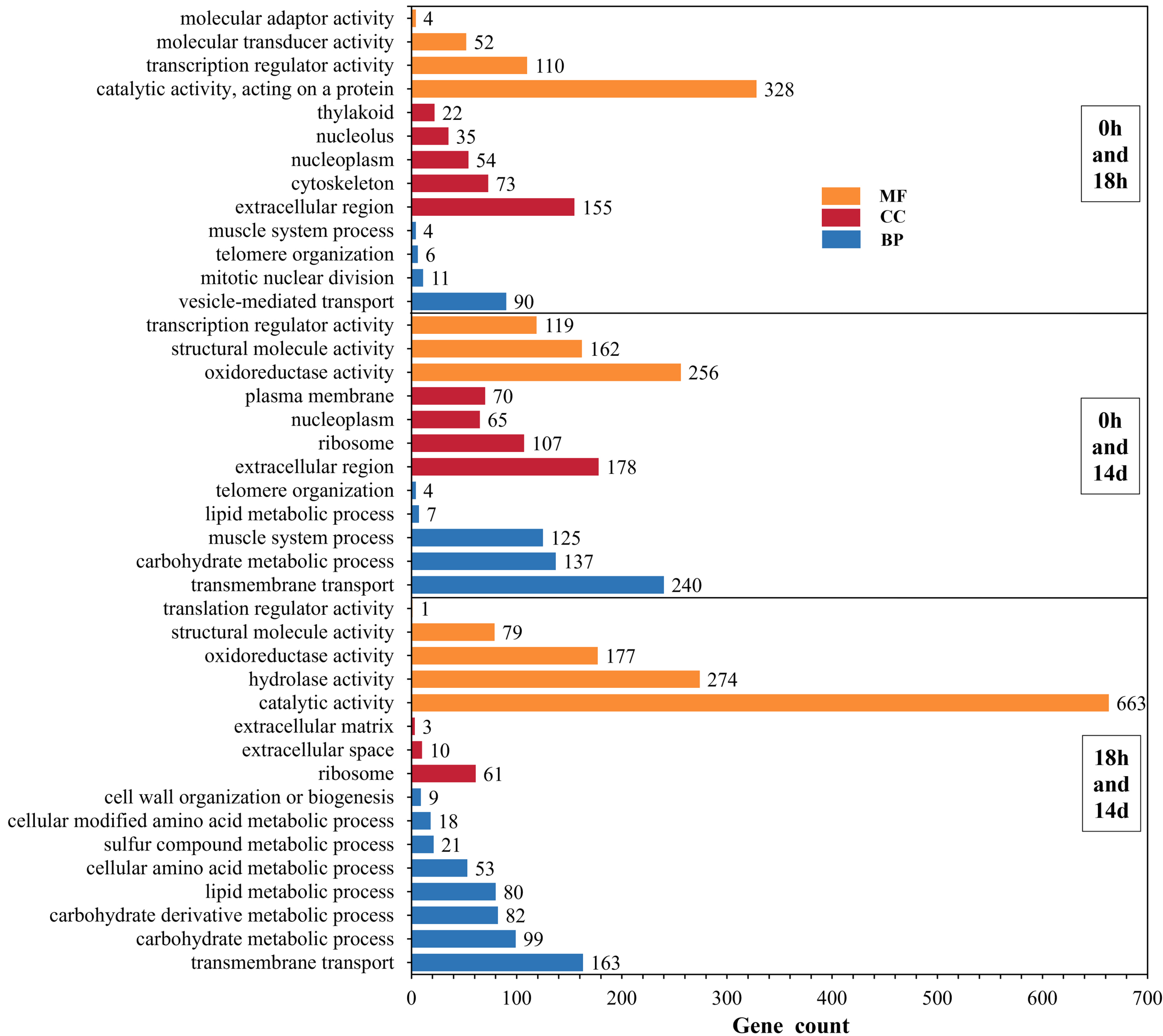

Supplement: Supplementary file 1 [file biology-14-00940-s001.zip › Figure S1.pdf]

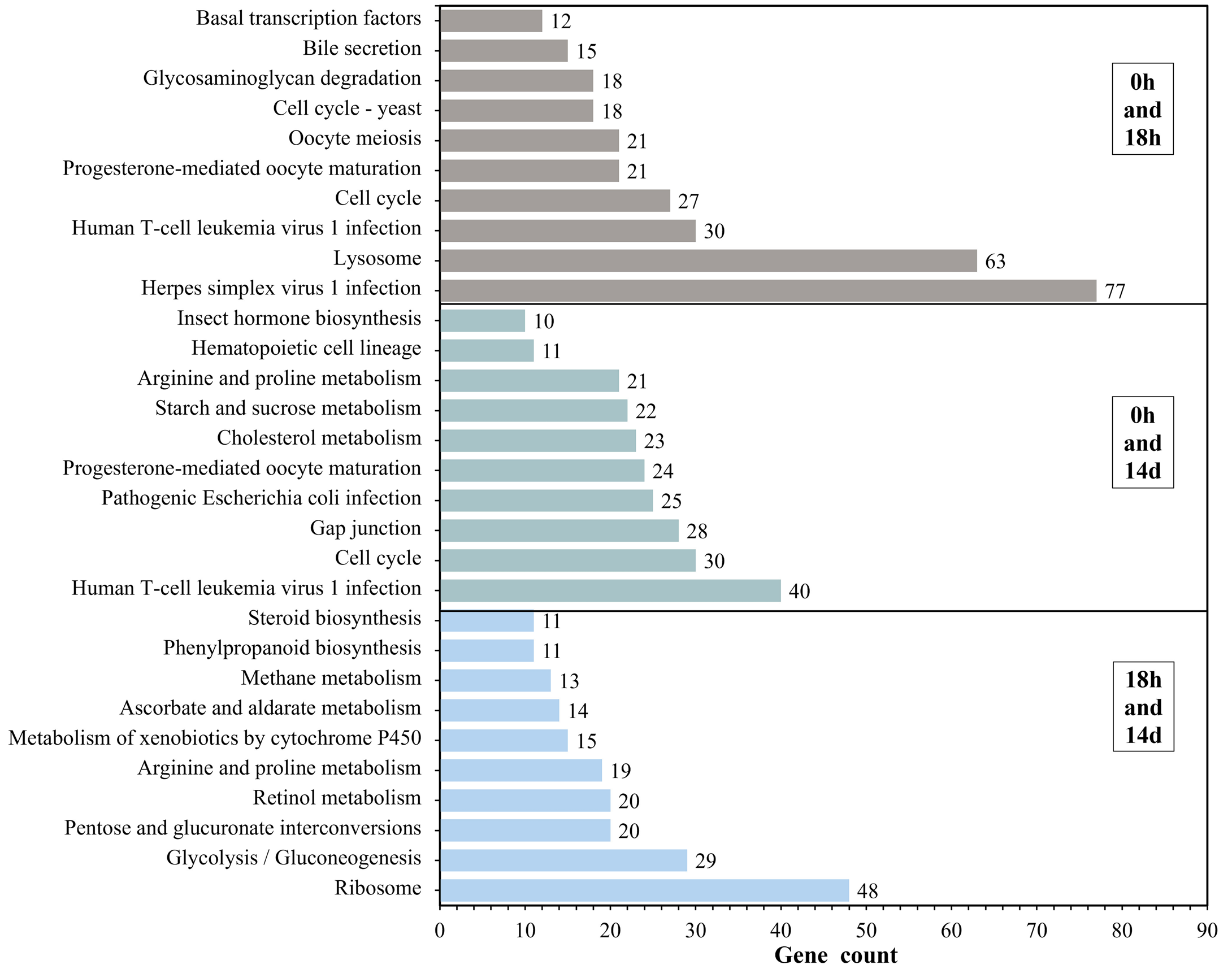

Supplement: Supplementary file 1 [file biology-14-00940-s001.zip › Figure S2.pdf]

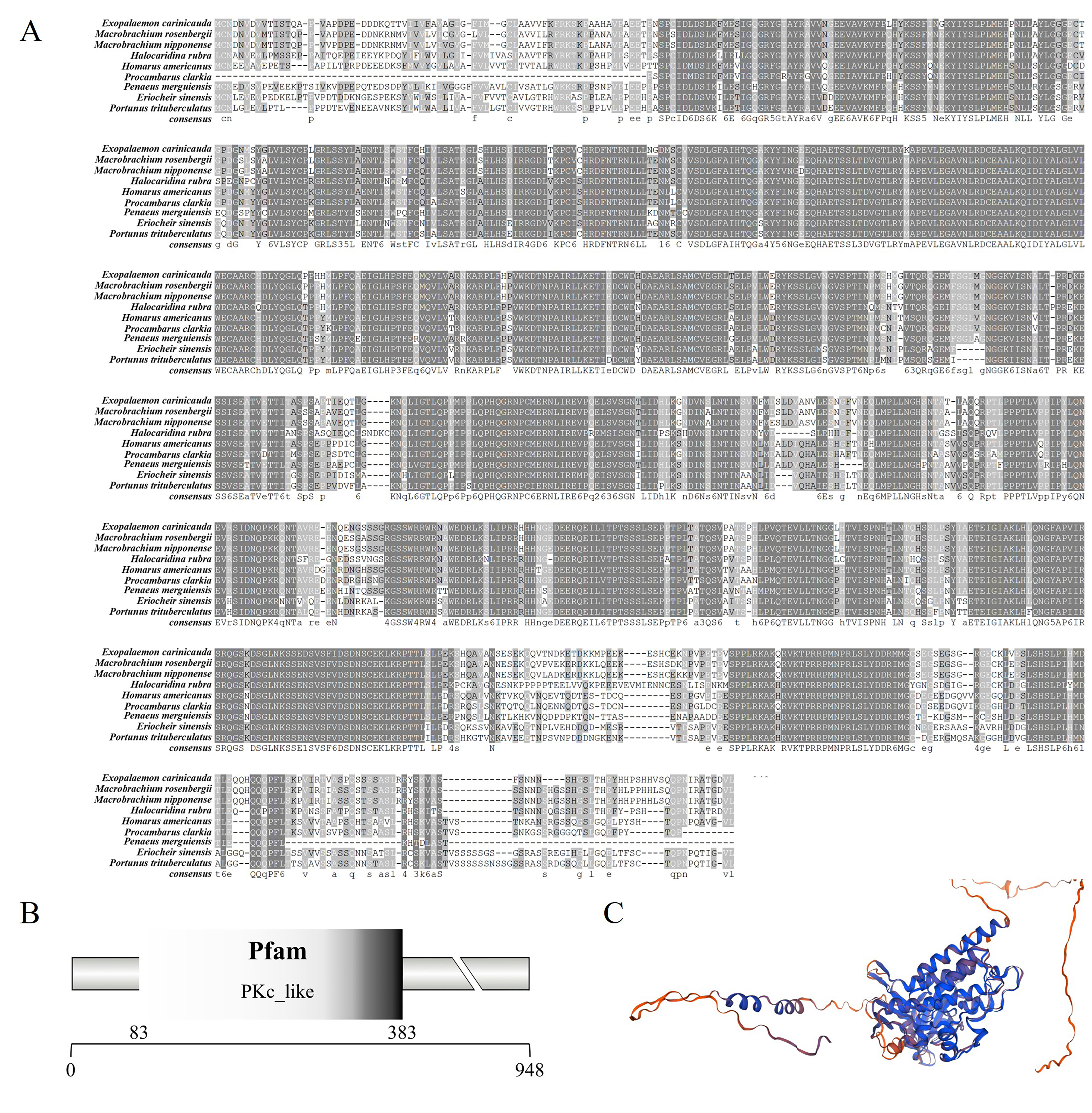

Supplement: Supplementary file 1 [file biology-14-00940-s001.zip › Figure S3.jpg]
